# Risk Stratification in Oral Cancer: A Novel Approach

**DOI:** 10.3389/fonc.2022.836803

**Published:** 2022-07-07

**Authors:** Irene Wen-Hui Tu, Nicholas Brian Shannon, Krishnakumar Thankappan, Deepak Balasubramanian, Vijay Pillai, Vivek Shetty, Vidyabhushan Rangappa, Naveen Hedne Chandrasekhar, Vikram Kekatpure, Moni Abraham Kuriakose, Arvind Krishnamurthy, Arun Mitra, Arun Pattatheyil, Prateek Jain, Subramania Iyer, Narayana Subramaniam, N. Gopalakrishna Iyer

**Affiliations:** ^1^ Department of Head and Neck Surgery Singapore General Hospital and National Cancer Centre Singapore and Duke-National University of Singapore (NUS) Medical School, Singapore, Singapore; ^2^ Department of Head and Neck Surgical Oncology, Amrita Institute of Medical Sciences, Amrita Vishwa Vidyapeetham, Kochi, India; ^3^ Department of Head and Neck Surgical Oncology, Mazumdar Shaw Medical Centre, Narayana Health, Bangalore, India; ^4^ Department of Surgical Oncology, Cancer Institute (WIA), Chennai, India; ^5^ Department of Head and Neck Surgical Oncology, Tata Medical Centre, Kolkata, India

**Keywords:** nomogram, cancer staging, head and neck tumors, oral squamous cell carcinoma (OSCC), pathological prognostic indicators, treatment escalation plan, overall survival

## Abstract

**Background:**

Oral squamous cell carcinoma (OSCC) is a common head and neck cancer with high morbidity and mortality. Currently, treatment decisions are guided by TNM staging, which omits important negative prognosticators such as lymphovascular invasion, perineural invasion (PNI), and histologic differentiation. We proposed nomogram models based on adverse pathological features to identify candidates suitable for treatment escalation within each risk group according to the National Comprehensive Cancer Network (NCCN) guidelines.

**Methods:**

Anonymized clinicopathologic data of OSCC patients from 5 tertiary healthcare institutions in Asia were divided into 3 risk groups according to the NCCN guidelines. Within each risk group, nomograms were built to predict overall survival based on histologic differentiation, histologic margin involvement, depth of invasion (DOI), extranodal extension, PNI, lymphovascular, and bone invasion. Nomograms were internally validated with precision–recall analysis and the Kaplan–Meier survival analysis.

**Results:**

Low-risk patients with positive pathological nodal involvement and/or positive PNI should be considered for adjuvant radiotherapy. Intermediate-risk patients with gross bone invasion may benefit from concurrent chemotherapy. High-risk patients with positive margins, high DOI, and a high composite score of histologic differentiation, PNI, and the American Joint Committee on Cancer (AJCC) 8th edition T staging should be considered for treatment escalation to experimental therapies in clinical trials.

**Conclusion:**

Nomograms built based on prognostic adverse pathological features can be used within each NCCN risk group to fine-tune treatment decisions for OSCC patients.

## Introduction

Oral squamous cell carcinoma (OSCC) is the most common histologic subtype of oral cancer (1), and the majority of head and neck squamous cell carcinoma arises from the oral cavity (2). It is associated with high morbidity and mortality, with a 5-year survival rate of only 50% (3). Since 1977, TNM staging guidelines by the American Joint Committee on Cancer (AJCC) and the Union for International Cancer Control (UICC) (1, 4), along with other adverse features, have been used to guide treatment decisions. In oral cancer, however, there has been little modification in the treatment philosophy for these patients over the past decade and a half (5). In spite of modifications in the staging system with the AJCC 8th edition (6), treatment protocols have not changed. It is unclear if prognostic factors such as depth of invasion (DOI) are an independent risk factor that requires adjuvant radiotherapy (7, 8) and if the standard of care for extranodal extension (ENE) continues to be adjuvant chemoradiotherapy (9).

Based on treatments recommended by the National Comprehensive Cancer Network (NCCN) guidelines (10), patients can be divided into three distinct groups: low, intermediate, and high risk. Surgical resection of the tumor with or without neck dissection is offered for low-risk patients, with the addition of radiotherapy for intermediate-risk patients and further addition of chemotherapy for high-risk patients. However, stratification within these groups has remained difficult; locoregional recurrence still occurs in one-third of treated early-stage OSCC despite clear surgical margins (11), while survival for advanced cancers (stages III/IV) is prognosticated solely based on nodal stage (6, 11).

TNM staging, although simple, leaves out multiple adverse pathological features such as lymphovascular invasion (LVI), perineural invasion (PNI), and histologic differentiation, which have been shown to be significant negative prognosticators in literature (12). TNM staging alone may be insufficient to fully address the needs for treatment selection and escalation (3). Models that assess the need for treatment escalation based on objective pathological features are still lacking, making treatment guidance clinically challenging.

Nomograms are user-friendly and effective prognostic models that can integrate a variety of clinical factors to provide improved precision in predicting survival outcomes (13). Current nomogram models described in the literature have attempted to predict prognosis after surgical resection (13), based on biomarkers (3) or only specific cancer types (11, 14). In this study, we looked at predictors of survival in each of the risk groups in order to derive nomograms that would help guide treatment, specifically to identify candidates who would potentially benefit from treatment escalation.

## Methods

### Patient Cohort

Demographic and clinicopathologic data of anonymized oral cancer patients were obtained from 5 tertiary healthcare institutions in Asia: Amrita Institute of Medical Sciences, Mazumdar Shaw Medical Centre, Tata Medical Center and Cancer Institute Adyar (India), and the National Cancer Center (Singapore) from 2006 to 2013. All patients were treatment-naive and underwent surgical resection. According to recommendations by an oral cancer multidisciplinary team within the respective institutions, some patients underwent further adjuvant therapy, which includes radiotherapy with or without chemotherapy. Not all patients were recommended adjuvant treatment.

Data collated include patient demographics, clinical and pathological disease features, treatments, and outcomes such as survival and recurrence. Subsites of oral cancer involved include the tongue, buccal, floor of the mouth, alveolus/retromolar, lip, and hard palate. Histologic differentiation was reported based on Broders’ grading system (4). PNI and LVI were reported positive when at least 33% of a nerve or vessel was noted to have any degree of tumor cell invasion (5). Histologic margin involvement was described as tumor involvement 0–5 mm from the margin or >5 mm from the margin. A positive margin was defined as tumor involvement 0–5 mm from the margin. ENE was recorded as the presence or absence of tumor spread beyond the tumor capsule, and DOI was measured as a continuous variable in mm. Gross cortical or medullary invasion of the maxilla or mandible was classified as “bone invasion.” All pathological reporting was performed by a dedicated head and neck pathologist in each institution and restaged according to the AJCC 8th edition pathological TNM staging for this study. Institutional ethics committee approval was obtained at all centers prior to data collection.

**Figure 1:**
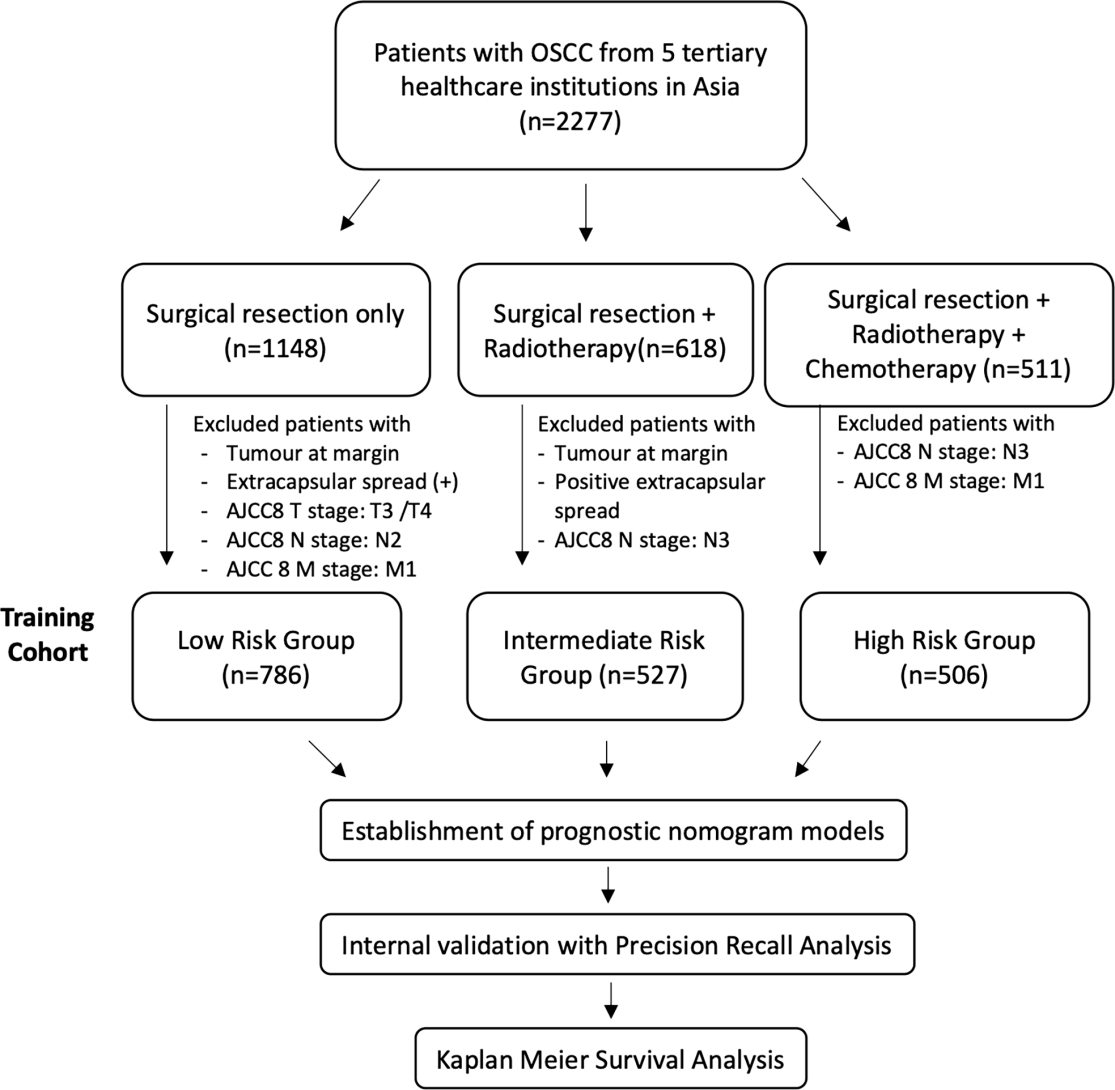
Flowchart of study cohort selection process.

The cohort was retrospectively divided into 3 groups according to treatment received based on the NCCN guidelines: “low-risk” patients were treated with surgery alone. According to NCCN guidelines, this should only be offered to patients with stage I/II (pT1–2) disease with no margin involvement and 0–1 node positive without ENE. However, in real-life medicine, the choice of therapy is confounded by patient factors, financial factors, and social factors. Thus, patients with tumors at the margin and extracapsular spread and AJCC8 T3/4, N2, and M1 patients were excluded, as illustrated in **Figure 1**. “Intermediate-risk” patients included patients who would have been recommended adjuvant radiotherapy after surgical resection according to NCCN guidelines: patients with stage III/IV disease who did not have positive margins or ENE. Patients with surgical resection and radiotherapy but had a tumor at the margin, positive extracapsular spread, and AJCC8 N3 disease were excluded. NCCN recommends that patients with positive margins and/or ENE should have adjuvant chemotherapy on top of surgical resection and adjuvant radiotherapy. Patients with surgery, adjuvant radiotherapy, and chemotherapy who had no (AJCC8) N3 or M1 disease were included in the “high-risk” group. Patients with (AJCC8) N3 or M1 are excluded from this study, as their tumors are not surgically resectable (**Figure 1**).

### Statistical Analysis

All statistical analyses were performed using the R program (Version 4.1.0), with the rms package (version 6.2.0) (15). Demographic data were described with univariate analysis. The primary end point was overall survival (OS), estimated according to the Kaplan–Meier method, which was defined as the time elapsed from the date of surgery to the date of death or last follow-up. The range of OS was defined as the upper 95% confidence limit minus the lower 95% confidence limit.

To screen for independent prognostic factors, Cox regression bivariate analysis of adverse clinicopathologic factors was performed. Adverse clinicopathologic factors analyzed for each subgroup include sex, age, margin, histologic differentiation, PNI, LVI, ENE, bone invasion, DOI, and AJCC8 TNM staging. p-Value and F-statistic were calculated for each variable. Variables with p-value <0.1 were used to build a Cox multivariable regression model using backward selection, and variables with p-value <0.05 were considered significant in multivariate analysis.

For each risk group, a nomogram was constructed reflecting all variables considered for multivariate analysis. All variables significant on bivariate analysis (p < 0.1) were included on the nomogram models if they exhibited a p-value <0.05 for prognosticating OS on the Kaplan–Meier analysis, even if they did not fulfill p < 0.05 on multivariate analysis. The Kaplan–Meier curves for OS were plotted for each variable selected for the nomogram. For each variable selected for the nomograms, their effect on OS was studied by Cox regression with a hazard ratio (HR) and p-value and illustrated with the Kaplan–Meier plots for each risk group (See **Supplementary Figures**).

For the high-risk group, in particular, a *post-hoc* analysis was conducted by stratifying high-risk patients based on a cumulative number of adverse histopathological features: a composite score of 1–4 was tabulated for PNI, histologic differentiation, and AJCC8 pathological T (pT) stages according to linear effect size based on variable coefficients (PNI, 0.1997; histologic differentiation, 0.2342; AJCC8 pT stage, 0.1171). Positive PNI and moderate/poorly differentiated cases were given 1 point each. Points were assigned to pT stages (AJCC8 criteria), as follows: T1 = 0, T2 = 1, and T3/4 = 2.

Each nomogram was internally validated with precision–recall analysis, as the size of each group varied widely. The predictive strength of each nomogram model was measured with the precision–recall area under the curve (PR AUC). Predictive performance was visualized using the Kaplan–Meier survival curves comparing modeled data on OS above and below a cutoff prediction score generated by a 5-year prediction model.

## Results

A retrospective analysis was performed on the demographic and clinicopathologic characteristics of 1,819 patients with oral cancers collected at 5 tertiary healthcare institutions from 2006 to 2013. Patients were divided into low-risk (n = 786), intermediate-risk (n = 527), and high-risk (n = 506) groups. The mean age was 54.7, 55.8, and 50.2 years for low-risk, intermediate-risk, and high-risk patients, respectively. Gender distribution was similar in the low-risk group (54.7% men), with a slight male predominance in the intermediate-risk (68.1% men) and high-risk (74.1% men) groups. Patients who used tobacco (smoked or chewed) were more prominently featured in the high-risk group (45.7%) compared to the low-risk (32.7%) and intermediate-risk (38.5%) groups. Subsite distribution of oral cancers studied included mostly the tongue, floor of the mouth, alveolus, and retromolar cancers. Clinical, demographic, and histopathological features in each subgroup are summarized in **Table 1**.

**Table 1:** Demographic and clinicopathologic profile of patients (n = 1,819).

	Low risk (n = 786)		Intermediate risk (n = 527)		High risk (n = 506)			
**Sex: male, n (%)**	422	(53.7)	359	(68.1)	375		(74.1)	
**Age, mean (SD)**	54.7	(13.7)	55.8	(12.3)	50.2		(11.8)	
**Smoker, n (%)**	257	(32.7)	203	(38.5)	231		(45.7)	
**Subsite, n (%)**								
**Tongue,**	492	(62.6)	208	(39.5)	283		(55.9)	
**Buccal mucosa**	66	(8.40)	75	(14.2)	51		(10.1)	
**Floor of Mouth**	160	(20.4)	132	(25.1)	119		(23.5)	
**Alveolus/retromolar**	47	(5.98)	94	(17.8)	43		(8.50)	
**Lip**	17	(2.16)	5	(0.949)	2		(0.395)	
**Hard palate**	3	(0.382)	11	(2.09)	6		(1.19)	
**Margin, mm, mean (SD)**	0.096	(0.298)	6.562	(3.743)	6.513		(3.512)	
**<5 mm from margin**	136	(17.3)	116	(22.0)	100		(19.8)	
**>5 mm from margin**	605	(77.0)	394	(74.8)	369		(72.9)	
**At margin**	0	(0)	0	(0)	30		(5.93)	
**Histologic differentiation, n (%)**								
**G1 (well differentiated)**	351	(44.7)	143	(27.1)	110		(21.7)	
**G2 (moderately differentiated)**	344	(43.8)	316	(60.0)	320		(63.2)	
**G3 (poorly differentiated)**	48	(6.11)	54	(10.2)	66		(13.0)	
**Diameter, mean (SD)**	18.2	(7.41)	33.7	(14.7)	1.95		(0.584)	
**Depth of invasion, mean (SD)**	6.45	(2.63)	14.5	(9.38)	18.7		(11.5)	
**(+) Perineural invasion, n (%)**	77	(9.80)	197	(37.4)	319		(63.0)	
**(+) Lymphovascular invasion, n (%)**	28	(3.56)	197	(37.4)	319		(63.0)	
**(+) Extracapsular spread, n (%)**	0	(0)	0	(0)	336		(66.4)	
**(+) Bone Invasion, n (%)**	0	(0)	152	(28.8)	121		(23.9)	
**Follow up time, mean (SD), years**	3.069	(2.594)	3.307	(3.487)	1.43		(1.386)	
**Death rate, n (%)**	14	(26.9)	99	(18.8)	85		(55.9)	
**Recurrence rate, n (%)**	143	(18.2)	135	(25.6)	205		(40.5)	
Overall survival^+^, months, median (range*)	37.9	(22.9)	17.0	(18.9)	22.7		(23.4)	

^+^Overall survival is defined as duration from date of surgery to last date of follow-up or death.

*Range is defined as upper 95% confidence limit–lower 95% confidence limit.

### Low-Risk Group

**Figure 2:**
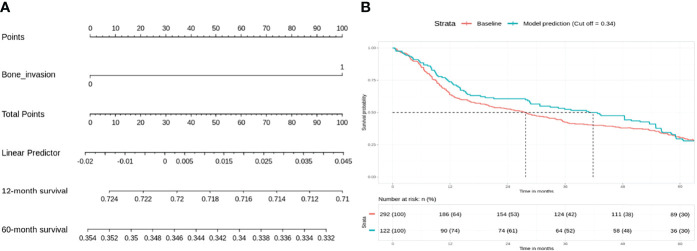
Low risk: nodal stage and perineural invasion (PNI) are negative prognosticators of overall survival. **(A)** Nomogram model. **(B)** Internal validation: prediction of 5‐year overall survival with nomogram model as compared to baseline (PR AUC = 0.800). PNI, perineural invasion; PR AUC, precision–recall area under the curve.

For the low-risk group, PNI (p = 0.062) and pathological nodal (pN) stage (p < 0.01) were negative prognostic factors for OS on bivariate analysis. Interestingly, multivariate Cox regression analysis revealed that only PNI was statistically significant in predicting poor OS (p < 0.01) in this group (**Table 2**). The Kaplan–Meier analysis for the pN stage revealed that it was significant in predicting poorer OS (HR = 1.96, p = 0.0622). The degree of impact of both PNI (HR = 25, p < 0.01) and pN stage was visualized succinctly in the nomogram to predict 12-month and 5-year survival, where a higher score depicted a poorer prognosis (**Figure 2A**). Internal validation of the nomogram model showed significant predictive strength as illustrated by **Figure 2B** (PR AUC = 0.800).

**Table 2:** Stepwise bivariate and multivariate Cox regression analysis for overall survival (p-value).

	Low risk		Intermediate risk		High risk	
	Bivariate	Multivariate	Bivariate	Multivariate	Bivariate	Multivariate
Sex	0.927		0.183		0.768	
Age	0.761		0.903		0.271	
Margin	0.685		0.935		0.010*	0.0213**
Histologic differentiation	0.258		0.627		0.033*	0.349
PNI (+)	0.062*	0.00683**	0.555		0.025*	0.0249**
LVI (+)	0.732		0.941		0.774	
ECS	-		-		0.865	
Bone invasion	-		0.031*	0.0313**	0.588	
DOI	0.991		0.640		0.003*	0.234
AJCC8 T stage	0.984		0.119		0.010*	0.351
AJCC8 N stage	0.009*	0.344	0.539		0.639	

PNI, perineural invasion; LVI, lymphovascular invasion; ECS, extracapsular spread; DOI, depth of invasion.

* p-value <0.1 (bivariate analysis).

** p-value <0.05 (multivariate analysis).

### Intermediate-Risk Group

**Figure 3:**
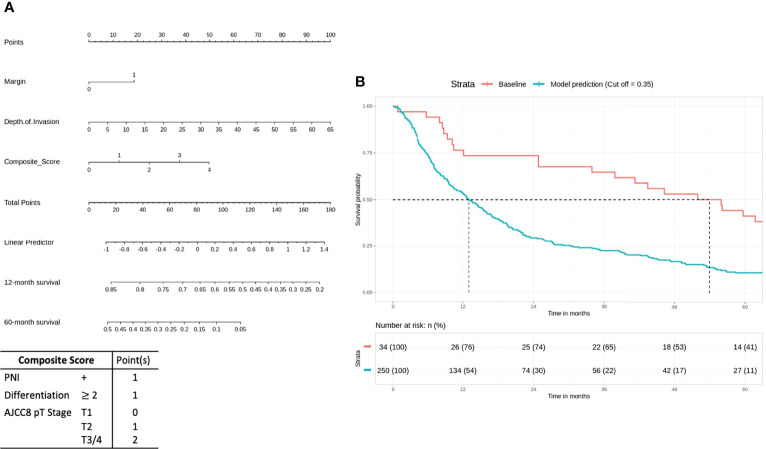
Intermediate-risk group: bone invasion is a significant negative prognosticator for overall survival (OS). **(A)** Nomogram model. **(B)** Internal validation: prediction of 5‐year overall survival with nomogram model as compared to baseline (PR AUC = 0.836). PR AUC, precision–recall area under the curve.

In the intermediate-risk group, only bone invasion (p < 0.05) was negatively prognostic for OS on bivariate analysis and remained a significant negative prognosticator on multivariate analysis (p = 0.0313). The nomogram for the intermediate-risk group (**Figure 3A**) illustrated the influence of bone invasion in predicting a negative prognosis for OS. Predictive strength was significant, with PR AUC = 0.836, on internal validation (**Figure 3B**).

### High-Risk Group

**Figure 4:**
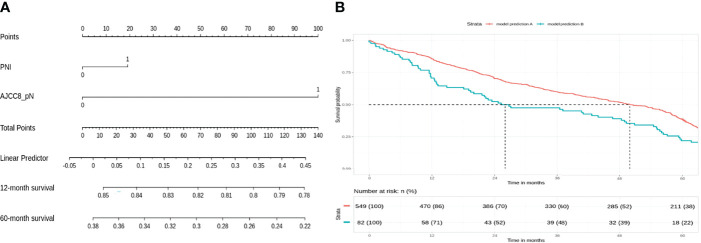
High-risk group: margin, depth of invasion, and a composite score are significant negative prognostic indicators for overall survival (OS). **(A)** Nomogram model. **(B)** Internal validation: prediction of 5‐year overall survival with nomogram model as compared to baseline (PR AUC = 0.925). Margin: 0, <5 mm from margin; 1, >5 mm from margin. Histologic differentiation: G1, well differentiated (1); G2, moderately differentiated (2); G3, poorly differentiated (3). PNI, perineural invasion; PR AUC, precision–recall area under the curve.

For the high-risk group, preliminary bivariate and multivariate analyses deemed margin, DOI, PNI, histologic differentiation, and pT staging to be statistically significant for poor prognosis (**Table 2**). However, the absolute impact on poor prognosis for the factors PNI, histologic differentiation, and pT staging was low compared to the factors margin (p = 0.0213) and DOI (p = 0.0249). A composite score of PNI, histologic differentiation, and pT staging was employed to better illustrate their impact. The Kaplan–Meier analysis showed that the composite score was significant for prognosticating poor OS (HR = 1.43, p < 0.01). Other negative prognostic factors reflected on the nomogram for high-risk patients include margin (HR = 2.07, p = 0.0134) and DOI (HR = 2.42, p < 0.01) (**Figure 4A**). Internal validation revealed significant predictive strength of PR AUC = 0.925 (**Figure 4B**).

## Discussion

Nomograms have been widely used to predict survival outcomes in a variety of cancers, including OSCC, by reflecting the prognostic strength of relevant variables (13). Previous nomograms designed for OSCC mostly focused on the prognostic effect of genes, biomarkers, or specific subsite and tumor grade of OSCC (3, 11, 13, 16). Recently, Zhou et al. published a nomogram model for risk stratification of OSCC based on clinical and pathological features (17). Montero et al. presented nomograms for preoperative prediction of OS and locoregional recurrence-free probability in OSCC (18). However, as cancer recurrence remained a significant problem after each step of treatment, there remained a need to develop a holistic prognostic model based on adverse pathological features commonly used in multidisciplinary tumor boards (MDTs) to better inform clinicians objectively on indications for treatment escalation.

The current practice for OSCC management is largely directed by multidisciplinary discussions in tumor boards, which take into consideration the cancer stage, adverse pathological factors, individual patient factors, and the likely functional consequences and morbidity of each treatment approach (19). Pathological staging by AJCC8 TNM staging is considered together with adverse pathological variables such as primary tumor site, histologic differentiation, perineural, lymphovascular, and bone invasion, as well as an ENE for prognostic risk stratification. As such, TNM staging alone remains insufficient in directing treatment decisions reliably (17).

In our study, we divided the multicentric patient population into 3 groups (low/intermediate/high) according to the NCCN. Patients were first divided according to the treatments they have been given and subsequently the training cohort was streamlined to ensure its characteristics reflect the prognostic risk profile of the relevant subgroup (**Figure 1**).

Patients in the low-risk group have localized (stage I/II) disease with 60%–80% 5-year OS (20). These patients were not advised of any adjuvant radiotherapy by the intra-institutional MDT. Our nomogram illustrated that positive nodal disease plays a major role in OS, even though the margins were clear post-resection. This was not unexpected and has been shown previously in National Cancer Database (NCDB) and Surveillance, Epidemiology, and End Results (SEER) data (21, 22) (**Figure 1**). However, PNI was unequivocally identified as a negative prognostic factor from Cox regression analysis. The strength of prognostic impact for PNI was marginal for OS (10 points ≤ 5% survival) on the nomogram. Multiple studies have stressed the importance of PNI as an independent negative prognostic factor associated with aggressive tumor behavior, tumor recurrence, and poor outcomes in T2N0 patients (23). However, other studies have suggested that adjuvant radiotherapy may not benefit this cohort of patients (24). LVI may not have been significant for survival in this cohort because a relatively small percentage of patients with LVI received solely surgical management (2.54%). It is likely that LVI was associated with a higher risk of nodal metastases, and these patients received adjuvant therapy as a result.

Patients in the intermediate risk group received adjuvant radiotherapy after surgical resection in view of locoregionally advanced disease and a higher risk of local recurrence (25). Our preliminary Cox regression analysis revealed that bone invasion had a significant impact on survival in this cohort. This is in concordance with existing literature, which identified bone invasion as an important prognostic factor to classify late-stage OSCC. It was associated with the worst quality of life and shorter life expectancy (26, 27) (**Figure 2**). This would suggest that there is likely a subset of patients with equivocal bone invasion who require adjuvant chemoradiotherapy; unfortunately, it was not possible to reclassify bone invasion in our cohort to better understand this. If there are other adverse features in addition to bone invasion, adjuvant chemotherapy use may be considered.

In the high-risk group that already received chemoradiotherapy, our analysis suggests that margins and DOI are important negative prognosticating factors, and the nomogram reflects a high impact on OS (50 points = 30% 2-year survival). The importance of DOI in OSCC is widely recognized, with the latest AJCC TNM classification incorporating DOI as part of the T staging (4). Positive margins are associated with tumor recurrence, diminished disease-specific survival, and poor OS even at 2 years (28). Interestingly, the impact of the ENE seems to be at least partially mitigated by adjuvant chemoradiotherapy. Our results also reveal that histologic differentiation, PNI, and T staging are independent negative prognosticators significant in both bivariate and multivariate Cox regression analysis. However, the prognostic value is weak individually, and a composite score of the 3 variables provides a stronger prognostic value for better visualization on the nomogram: the T stage relates to the anatomical extent of tumor growth and invasion into surrounding tissues and is an important prognostic factor in AJCC8 TNM staging (4). Chen et al. showed that poor tumor differentiation is a negative predictor of subsequent distant metastasis in locoregionally advanced OSCC (29), while Jardim et al. argued for the prognostic impact of PNI in advanced-stage OSCC (30). While literature suggested that each of these 3 factors are important adverse pathological prognosticators, in the context of high-risk OSCC, their impact on predicting poorer prognosis was marginal independently. The prognostic significance of the composite score; however, suggests that their impact should not be overlooked. Therefore, our analysis suggests that positive margin, DOI, and a composite score of histologic differentiation, PNI, and T stage (AJCC8) are important negative prognostic indicators for poor survival.

The limitations of this study include, first, the heterogeneity of OSCC as tumor characteristics differed significantly across subsites. Yet universal treatment guidelines such as TNM staging had been able to provide sound treatment advice on OSCC as a whole, and our study largely revolved around known adverse pathological parameters common to all subsites of OSCC. Second, we assumed that treatment decisions at each center were based only on the pathology of the disease and did not consider patient factors such as finances. Third, all recommendations were made based on statistical modeling, which was co-relational and hypothesis-generating in nature. Causation relationships between the variables and OS would require further randomized control trials to be firmly established. The inclusion of molecular markers for prognostication in this group may have added value but unfortunately was beyond the scope of this work. Fourth, pathological interpretation of DOI and extracapsular invasion may defer across different pathologists across different nations. In this study, we opted to record these variables objectively without further interpretation: presence or absence of extracapsular invasion(,) and the objective measurement of the DOI in mm directly. Finally, our database was collected postoperatively and had limited preoperative factors to build an Asia-specific preoperative nomogram to supplement our postoperative nomograms. This could be explored in future studies. Further work could also look into prognostication with specifically medullary invasion (31), instead of overall bone invasion.

By constructing these nomograms based on real-world data of patients who received appropriate adjuvant therapy after surgical resection, it was our intention to determine specific cohorts who had a higher risk of death and hence may be candidates for treatment escalation. To our knowledge, this is the first report of its kind. Our findings recommend that low-risk patients with positive pathological nodal involvement and/or positive PNI should consider adjuvant radiotherapy. Intermediate-risk patients with gross bone invasion may benefit from concurrent chemotherapy; however, it is likely that this decision needs to be taken on a patient-by-patient basis after consideration of other adverse features and the extent of bone invasion. Finally, high-risk patients with positive margins, high DOI, and a high composite score of histologic differentiation, PNI, and AJCC8 T staging should be considered for treatment escalation or experimental therapies in the setting of clinical trials.

## Data Availability Statement

The data analyzed in this study is subject to the following licenses/restrictions: This dataset was built from information on patient details that have been de-identified. However as these details were originally from patients, they cannot be available publicly. Please direct any request for data to the corresponding author. Requests to access these datasets should be directed to NI, gmsngi@duke.nus.sg.

## Author Contributions

IT and NS are co first authors contributing equally to the conception, design of study and data analysis. KT, DB, VP, VS, VR, NC, VK, MK, AK, AM, AP, PJ, and SI contributed the dataset of study used for training and internal validation of the proposed nomogram model. NI and NS are both corresponding authors contributing to conception and design of study. All authors contributed to manuscript revision, read, and approved the submitted version.

## Conflict of Interest

The authors declare that the research was conducted in the absence of any commercial or financial relationships that could be construed as a potential conflict of interest.

## Publisher’s Note

All claims expressed in this article are solely those of the authors and do not necessarily represent those of their affiliated organizations, or those of the publisher, the editors and the reviewers. Any product that may be evaluated in this article, or claim that may be made by its manufacturer, is not guaranteed or endorsed by the publisher.

## References

[1] Almangush A Pirinen M Youssef O Mäkitie AA Leivo I. Risk Stratification in Oral Squamous Cell Carcinoma Using Staging of the Eighth American Joint Committee on Cancer: Systematic Review and Meta-Analysis. Head Neck (2020) 42(10):3002–17. doi: 10.1002/hed.26344 32548858

[2] Chi AC Day TA Neville BW. Oral Cavity and Oropharyngeal Squamous Cell Carcinoma—an Update. CA: Cancer J Clin (2015) 65(5):401–21. 10.3322/caac.21293 26215712

[3] Huang Z-D Yao Y-Y Chen T-Y Zhao Y-F Zhang C Niu Y-M. Construction of Prognostic Risk Prediction Model of Oral Squamous Cell Carcinoma Based on Nine Survival-Associated Metabolic Genes. Front Physiol (2021) 12:290. doi: 10.3389/fphys.2021.609770 PMC8011568 33815132

[4] Lydiatt WM Patel SG O'Sullivan B Brandwein MS Ridge JA Migliacci JC. Head and Neck Cancers—Major Changes in the American Joint Committee on Cancer Eighth Edition Cancer Staging Manual. CA: Cancer J Clin (2017) 67(2):122–37. 10.3322/caac.21389 28128848

[5] Almangush A Mäkitie AA Triantafyllou A de Bree R Strojan P Rinaldo A. Staging and Grading of Oral Squamous Cell Carcinoma: An Update. Oral Oncol (2020) 107:104799. doi: 10.1016/j.oraloncology.2020.104799 32446214

[6] Mattavelli D Ferrari M Taboni S Morello R Paderno A Rampinelli V. The 8th TNM Classification for Oral Squamous Cell Carcinoma: What Is Gained, What is Lost, and What Is Missing. Oral Oncol (2020) 111:104937. doi: 10.1016/j.oraloncology.2020.104937 32750558

[7] Ebrahimi A Gil Z Amit M Yen TC t. Liao C Chaturvedi P. Depth of Invasion Alone as an Indication for Postoperative Radiotherapy in Small Oral Squamous Cell Carcinomas: An International Collaborative Study. Head Neck (2019) 41(6):1935–42. doi: 10.1002/hed.25633 PMC6563806 30801885

[8] Subramaniam N Balasubramanian D Murthy S Rathod P Vidhyadharan S Thankappan K. Impact of Postoperative Radiotherapy on Survival and Loco-Regional Control in Node-Negative Oral Cavity Tumours Classified as T3 Using the AJCC Cancer Staging Manual Eighth Edition. Int J Oral Maxillofac Surg (2019) 48(2):152–6. doi: 10.1016/j.ijom.2018.07.009 30243830

[9] Bernier J Cooper JS Pajak T Van Glabbeke M Bourhis J Forastiere A. Defining Risk Levels in Locally Advanced Head and Neck Cancers: A Comparative Analysis of Concurrent Postoperative Radiation Plus Chemotherapy Trials of the EORTC (# 22931) and RTOG (# 9501). Head Neck: J Sci Specialties Head Neck (2005) 27(10):843–50. doi: 10.1002/hed.20279 16161069

[10] Colevas AD Yom SS Pfister DG Spencer S Adelstein D Adkins D. NCCN Guidelines Insights: Head and Neck Cancers, Version 1.2018. J Natl Compr Cancer Netwo (2018) 16(5):479–90. doi: 10.6004/jnccn.2018.0026 29752322

[11] Balasubramanian D Subramaniam N Missale F Marchi F Dokhe Y Vijayan S. Predictive Nomograms for Oral Tongue Squamous Cell Carcinoma Applying the American Joint Committee on Cancer/Union Internationale Contre Le Cancer 8th Edition Staging System. Head Neck (2021) 43(4):1043–55. doi: 10.1002/hed.26554 33529403

[12] Subramaniam N Balasubramanian D Murthy S Limbachiya S Thankappan K Iyer S. Adverse Pathologic Features in Early Oral Squamous Cell Carcinoma and the Role of Postoperative Radiotherapy—A Review. Oral surg Oral medic Oral Pathol Oral Radiol (2017) 124(1):24–31. doi: 10.1016/j.oooo.2017.03.002 28506568

[13] Nie Z Zhao P Shang Y Sun B. Nomograms to Predict the Prognosis in Locally Advanced Oral Squamous Cell Carcinoma After Curative Resection. BMC Cancer (2021) 21(1):1–17. doi: 10.1186/s12885-021-08106-x 33827452 PMC8028060

[14] Zheng S Yang J Li C Han D Xu F Elishilia Kaaya R. Prognostic Exploration of All-Cause Death in Gingival Squamous Cell Carcinoma: A Retrospective Analysis of 2076 Patients. J Oncol 2021(2021). doi: 10.1155/2021/6676587 PMC8019369 33854548

[15] R. C. Team. R: A Language and Environment for Statistical Computing (2021). Available at: https://www.R-project.org/.

[16] Wang S Li T Liu H Wei W Yang Y Wang C. A Combined Prediction Model for Lymph Node Metastasis Based on a Molecular Panel and Clinicopathological Factors in Oral Squamous Cell Carcinoma. Front Oncol (2021) 11:1211. doi: 10.3389/fonc.2021.660615 PMC8100439 33968767

[17] Zhou J Li H Cheng B Cao R Zou F Yang D. Derivation and Validation of a Prognostic Scoring Model Based on Clinical and Pathological Features for Risk Stratification in Oral Squamous Cell Carcinoma Patients: A Retrospective Multicenter Study. Front Oncol (2021) 11:1500. doi: 10.3389/fonc.2021.652553 PMC8195273 34123806

[18] Montero PH Yu C Palmer FL Patel PD Ganly I Shah JP. Nomograms for Preoperative Prediction of Prognosis in Patients With Oral Cavity Squamous Cell Carcinoma. Cancer (2014) 120(2):214–21. doi: 10.1002/cncr.28407 24399417

[19] Wheless SA McKinney KA Zanation AM. A Prospective Study of the Clinical Impact of a Multidisciplinary Head and Neck Tumor Board. Otolaryngol—Head Neck Surg (2010) 143(5):650–4. doi: 10.1016/j.otohns.2010.07.020 PMC2994101 20974334

[20] Viet CT Yu G Asam K Thomas CM Yoon AJ Wongworawat YC. The REASON Score: An Epigenetic and Clinicopathologic Score to Predict Risk of Poor Survival in Patients With Early Stage Oral Squamous Cell Carcinoma. biomark Res (2021) 9(1):1–13. doi: 10.1186/s40364-021-00292-x 34090518 PMC8178935

[21] Shrime MG Gullane PJ Dawson L Kim J Gilbert RW Irish JC. The Impact of Adjuvant Radiotherapy on Survival in T1-2N1 Squamous Cell Carcinoma of the Oral Cavity. Arch Otolaryngol–Head Neck Surg (2010) 136(3):225–8. doi: 10.1001/archoto.2010.22 20231637

[22] Chen MM Harris JP Hara W Sirjani D Divi V. Association of Postoperative Radiotherapy With Survival in Patients With N1 Oral Cavity and Oropharyngeal Squamous Cell Carcinoma. JAMA Otolaryngol–Head Neck Surg (2016) 142(12):1224–30. doi: 10.1001/jamaoto.2016.3519 27832255

[23] Cheng C-S Chen C-C Liu Y-C Wang C-C Chou Y-S. Peri-Neural Invasion is An Important Prognostic Factor of T2N0 Oral Cancer. (2021). ResearchSquare, Taiwan (ROC). 10.3390/medicina58121809 PMC9787494 36557011

[24] Bur AM Lin A Weinstein GS. Adjuvant Radiotherapy for Early Head and Neck Squamous Cell Carcinoma With Perineural Invasion: A Systematic Review. Head Neck (2016) 38(S1):E2350–7. doi: 10.1002/hed.24295 26613965

[25] Stenson KM Brockstein BE. Shiyu Song: Overview of Treatment for Head and Neck Section: Management of SCC - Localized (Early Stage Disease). In: Fried MP Posner MR Brizel DM Shah S, editors. UpToDate (2021). Available at: www.uptodate.com.

[26] Brown JS Lowe D Kalavrezos N D'Souza J Magennis P Woolgar J. Patterns of Invasion and Routes of Tumor Entry Into the Mandible by Oral Squamous Cell Carcinoma. Head Neck: J Sci Specialties Head Neck (2002) 24(4):370–83. doi: 10.1002/hed.10062 11933179

[27] Yao CM Chang EI Lai SY. Contemporary Approach to Locally Advanced Oral Cavity Squamous Cell Carcinoma. Curr Oncol Rep (2019) 21(11):1–9. doi: 10.1007/s11912-019-0845-8 31701240

[28] Jain PV Sharan R Manikantan K Clark GM Chatterjee S Mallick I. Redefining Adequate Margins in Oral Squamous Cell Carcinoma: Outcomes From Close and Positive Margins. Eur Arch Oto-Rhino-Laryngol (2020) 277(4):1155–65. doi: 10.1007/s00405-019-05779-w 31897720

[29] Chen T-C Hsu C-W Lou P-J Ko J-Y Yang T-L Chen C-N. The Clinical Predictive Factors for Subsequent Distant Metastasis in Patients With Locoregionally Advanced Oral Squamous Cell Carcinoma. Oral Oncol (2013) 49(4):367–73. doi: 10.1016/j.oraloncology.2012.10.006 23142556

[30] Jardim J Francisco A Gondak R Damascena A Kowalski L. Prognostic Impact of Perineural Invasion and Lymphovascular Invasion in Advanced Stage Oral Squamous Cell Carcinoma. Int J Oral Maxillofac Surg (2015) 44(1):23–8. doi: 10.1016/j.ijom.2014.10.006 25457832

[31] Ebrahimi A Murali R Gao K Elliott MS Clark JR. The Prognostic and Staging Implications of Bone Invasion in Oral Squamous Cell Carcinoma. Cancer (2011) 117(19):4460–7. doi: 10.1002/cncr.26032 21437887

