# Supplementary material for: Risk Stratification in Oral Cancer: A Novel Approach
Source: Front Oncol. 2022 Jul 7;12:836803. doi: 10.3389/fonc.2022.836803 (PMC9301677; doi:10.3389/fonc.2022.836803)

**Supplementary Figure 1.** Low Risk: Nodal stage and perineural invasion (PNI) are negative prognosticators of overall survival (OS)


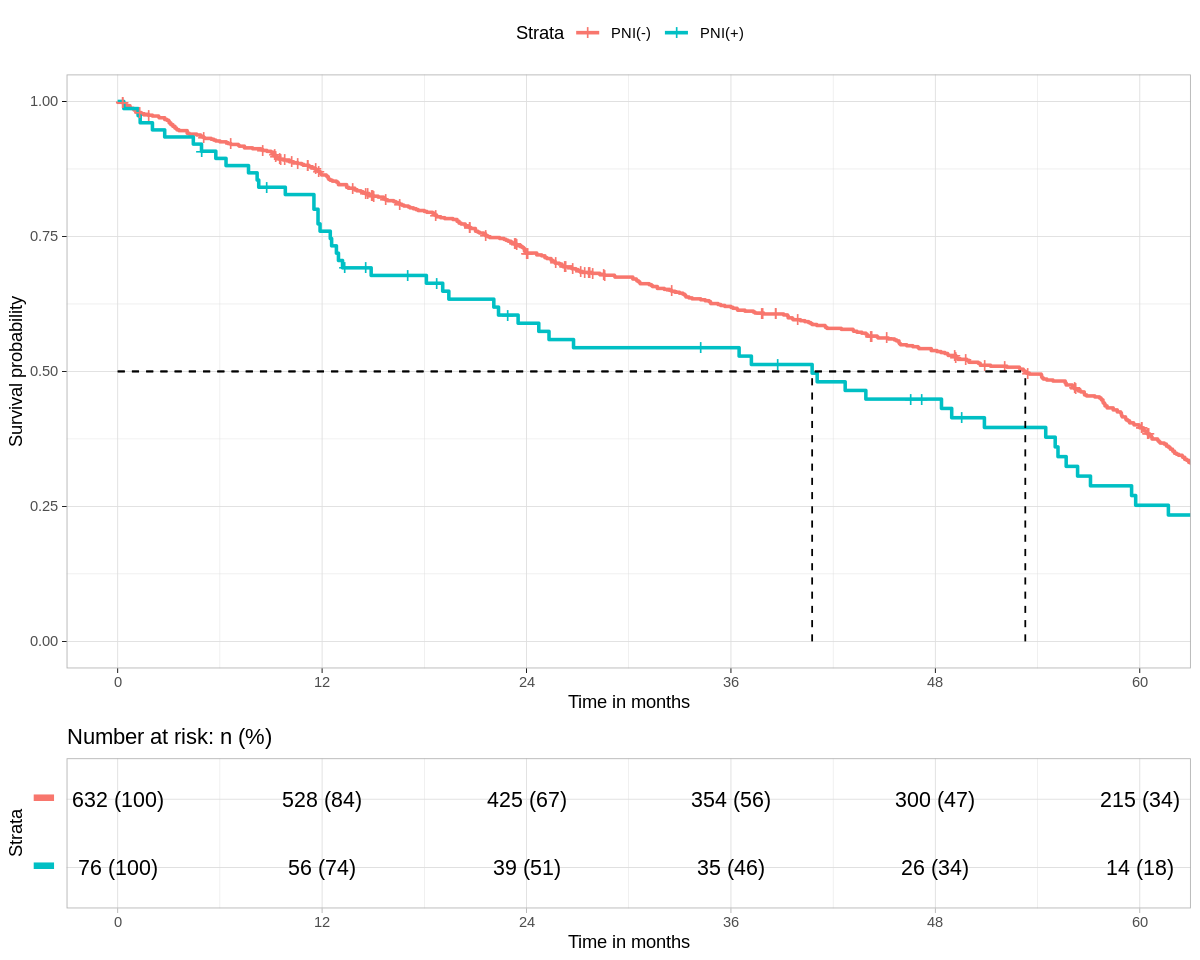

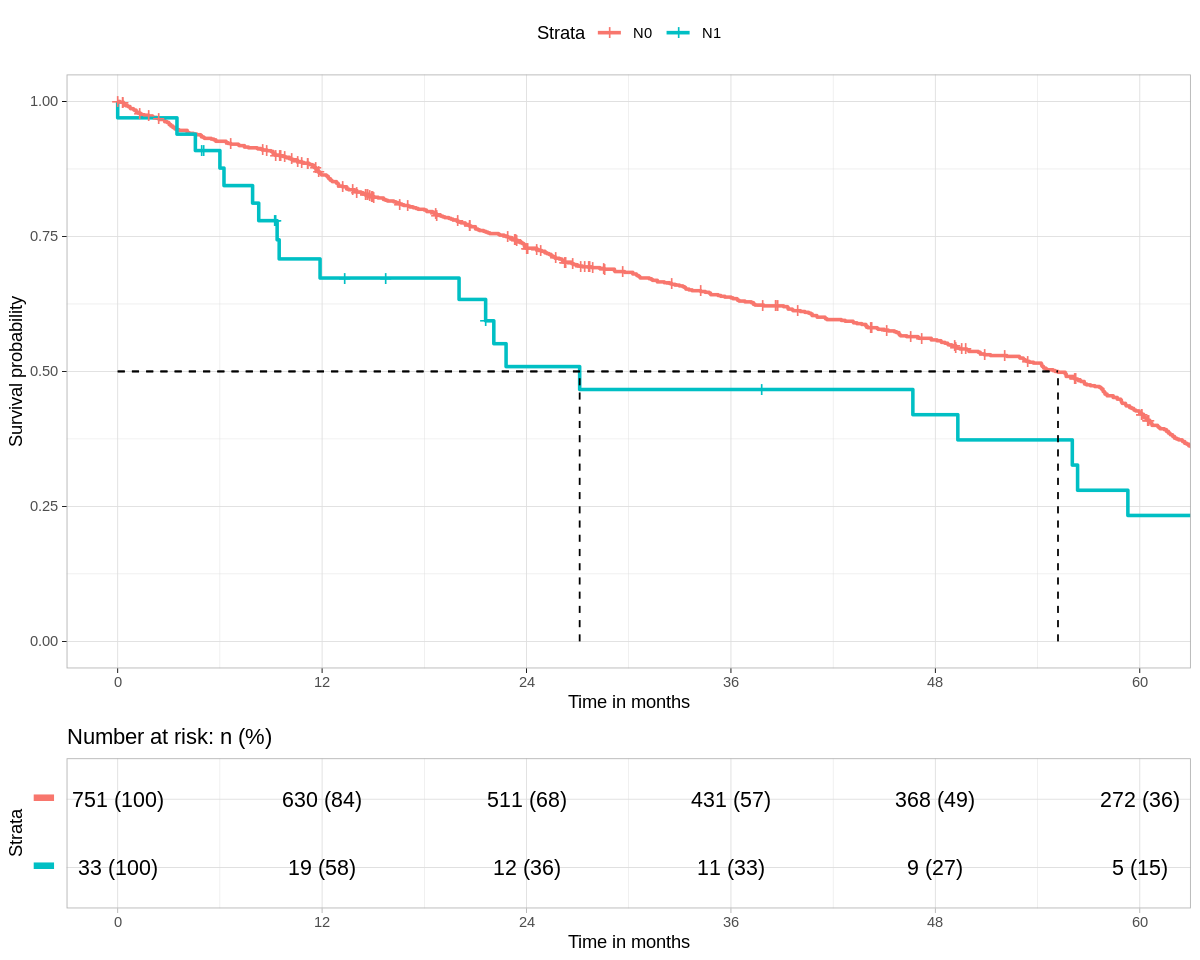


(a) Kaplan Meier Analysis: Effect of perineural invasion (PNI) on OS

Hazard Ratio: 25.0

p-value: 0.00859


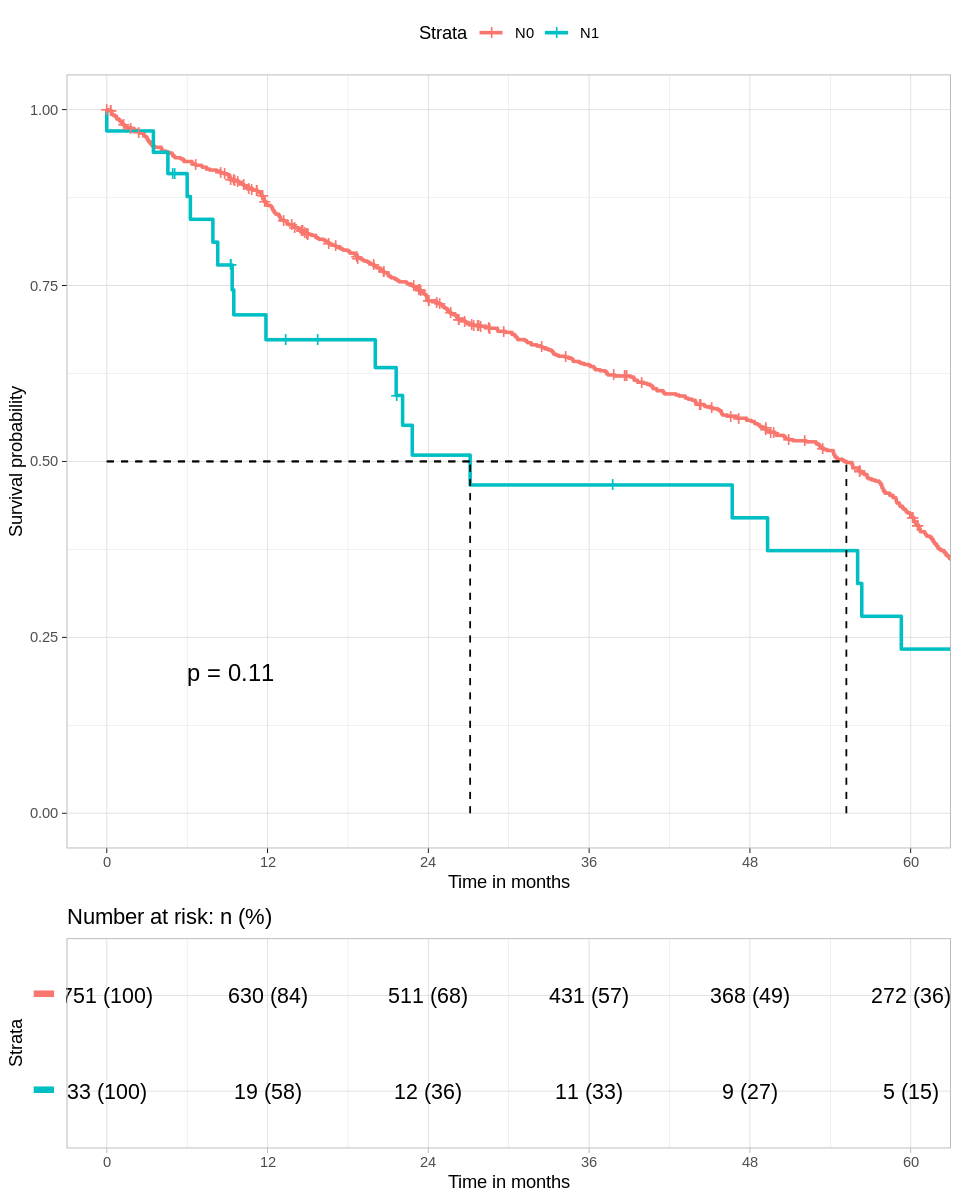


Hazard Ratio: 1.96

p-value: 0.0622


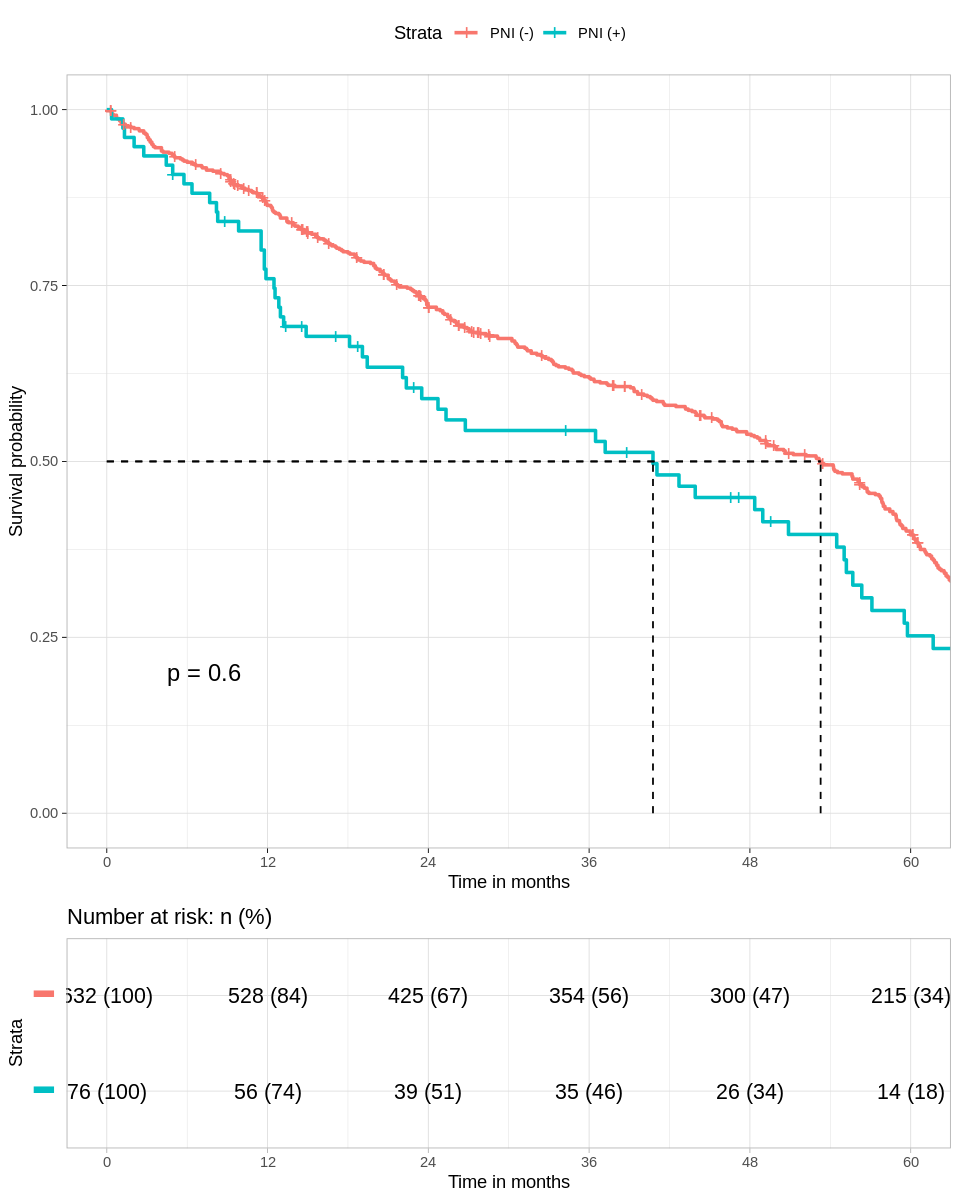


(b) Kaplan Meier Analysis: Effect of nodal stage (AJCC8) on OS

**Supplementary Figure 2.** Intermediate Risk Group: Kaplan Meier plot showing effect of bone invasion on overall survival (OS)


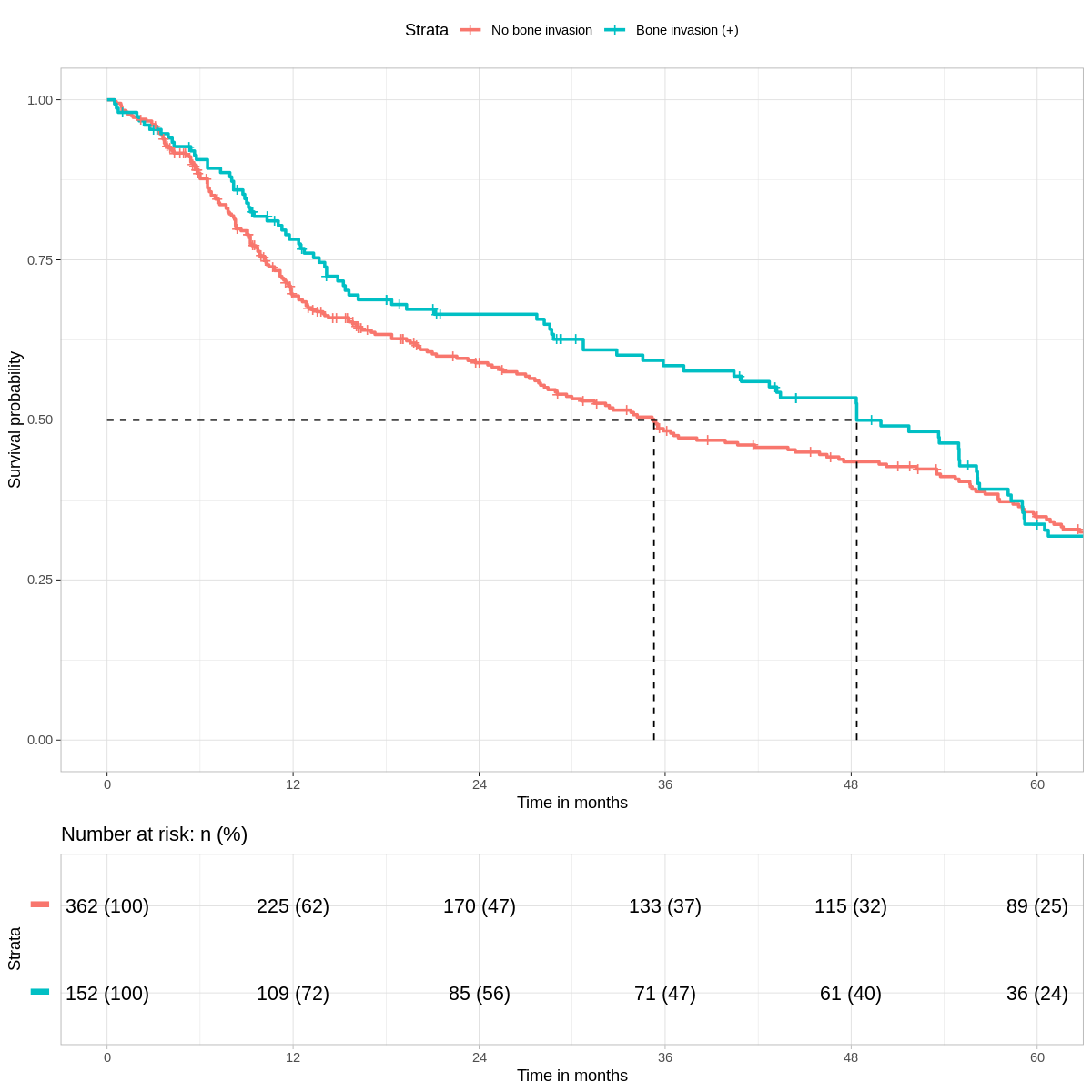


Hazard Ratio: 2.22

p-value: 0.0313


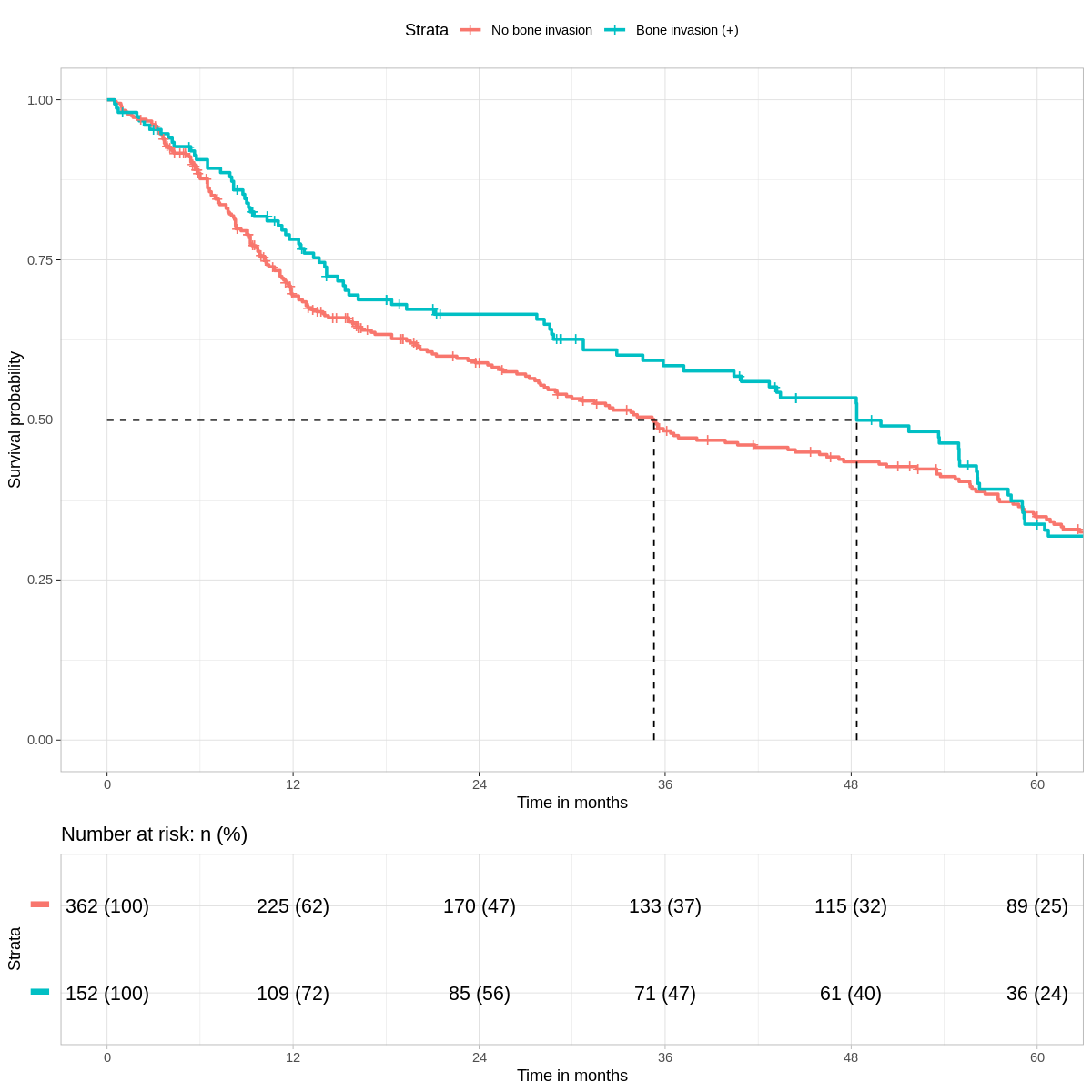


**Supplementary Figure 3.** High Risk Group: Margin, depth of invasion and a composite score are significant negative prognostic indicators for overall survival (OS)


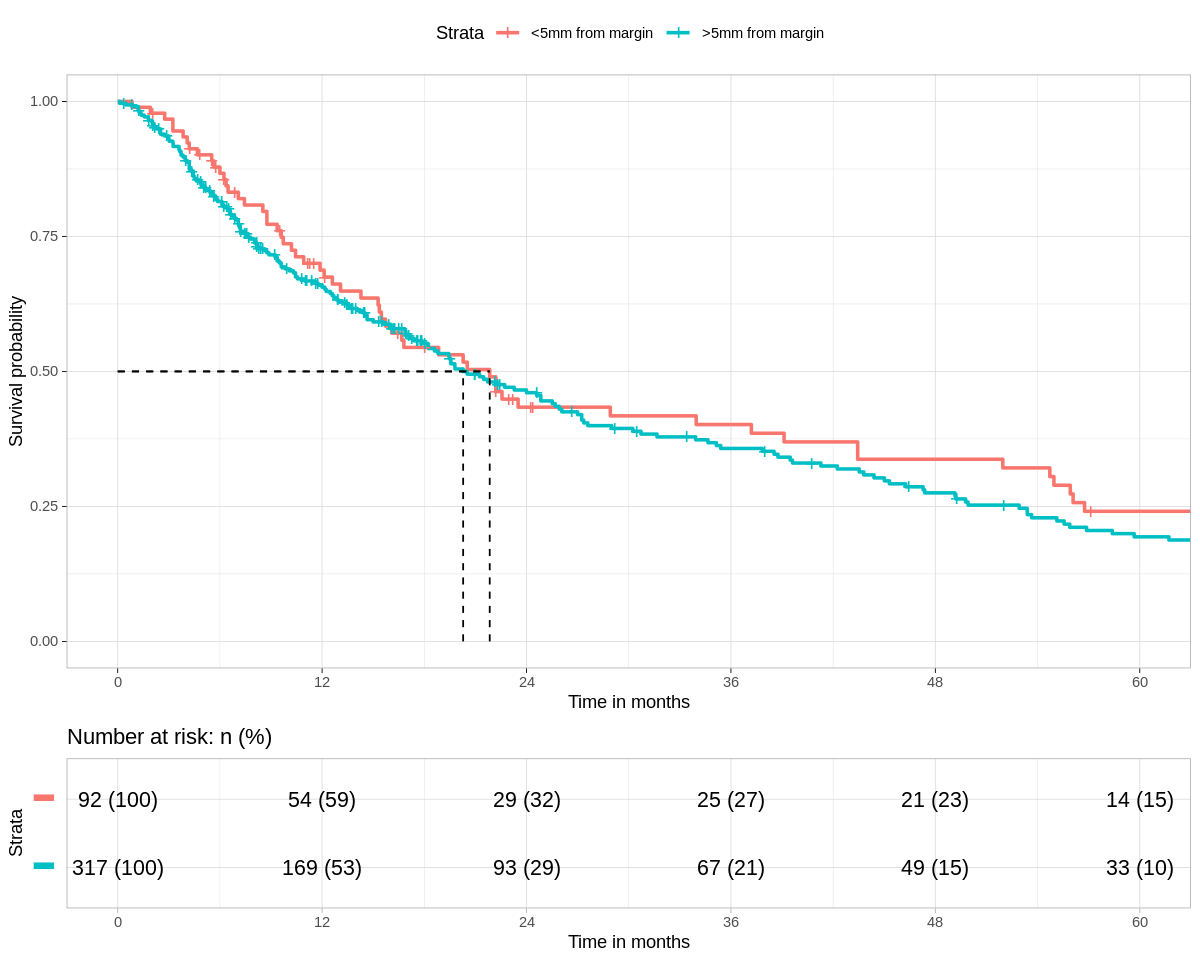


Hazard Ratio: 2.97

p-value: 0.0134


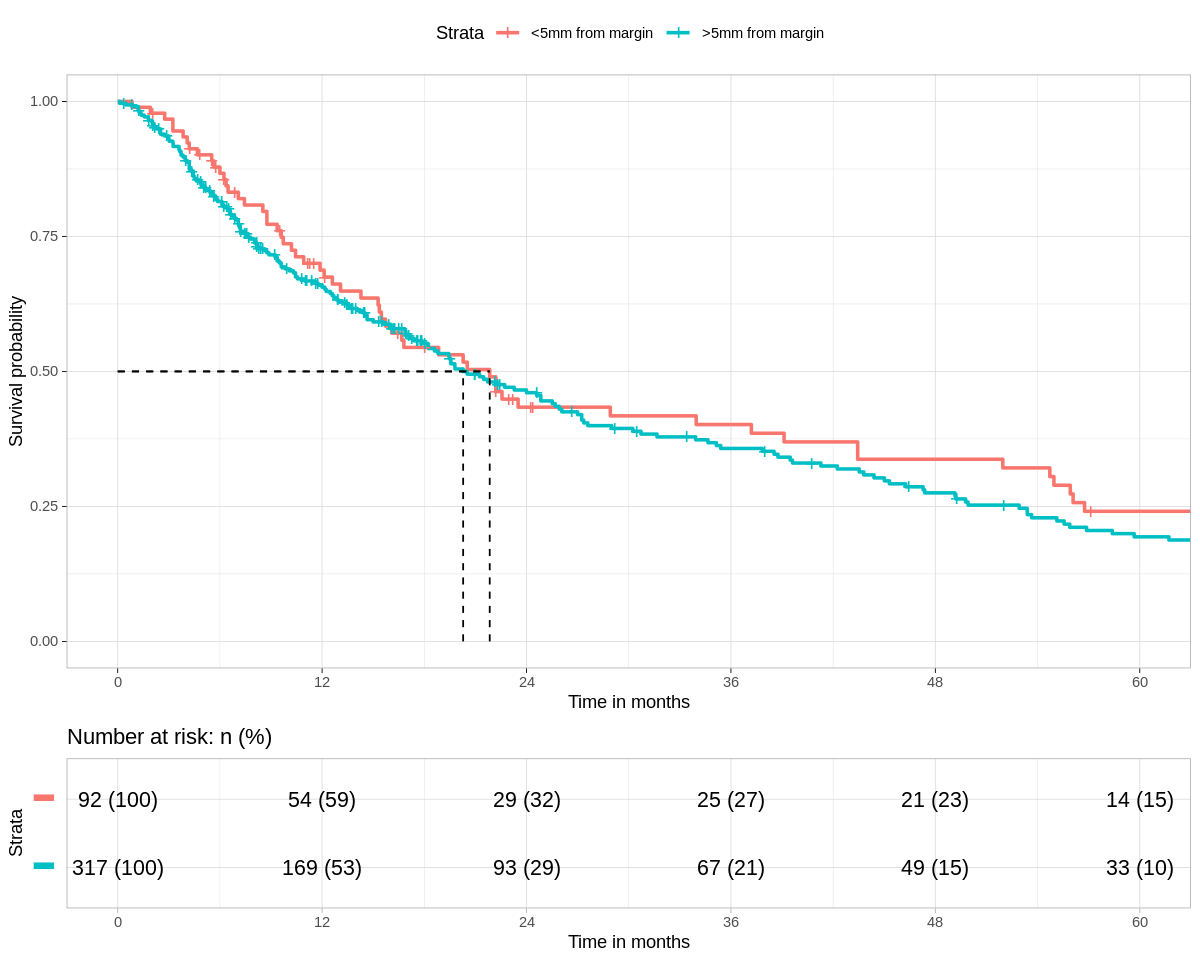


(b) Kaplan Meier Analysis: Effect of Composite Score on OS

(a) Kaplan Meier Analysis: Effect of Tumour Margin on OS


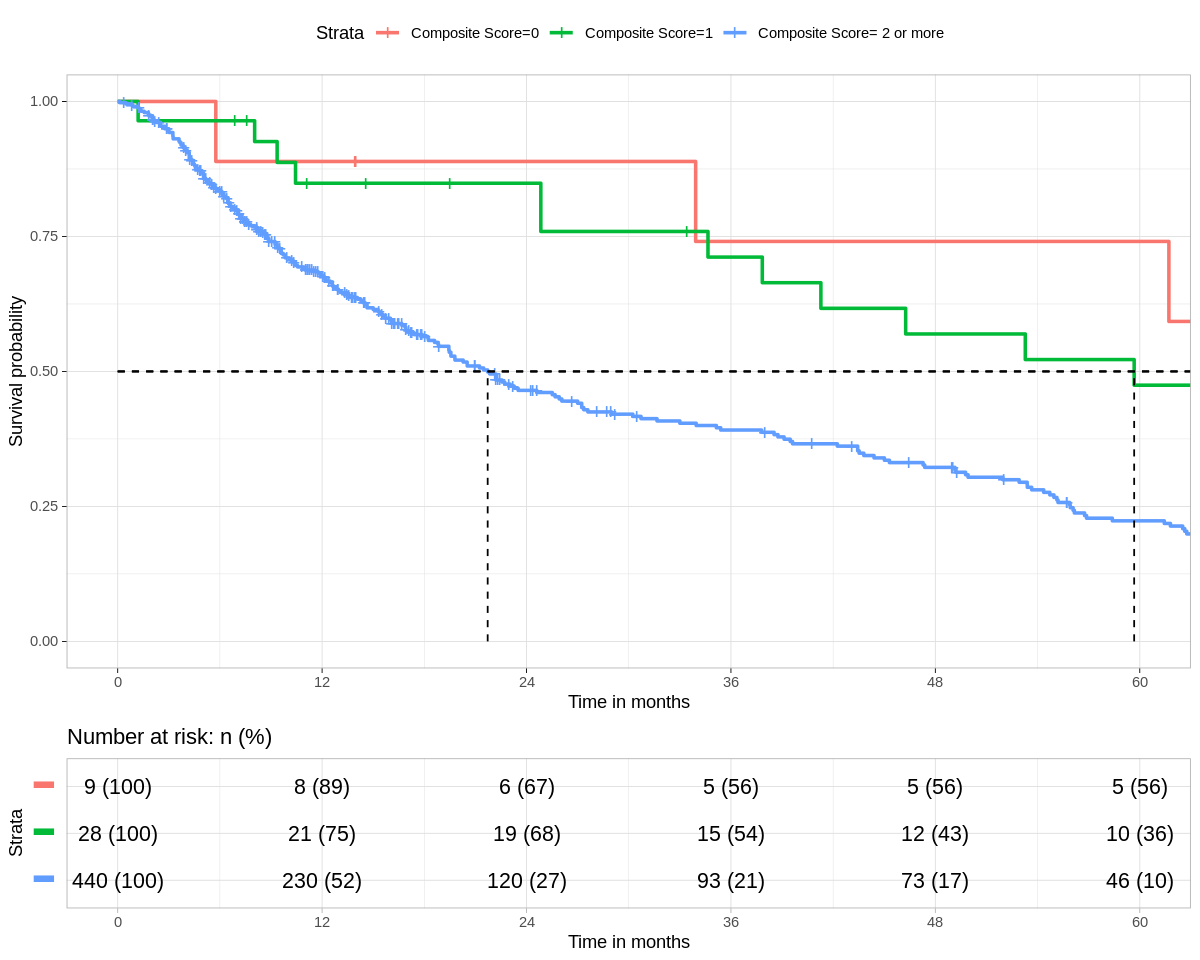


Hazard Ratio: 1.43

p-value: 0.00409


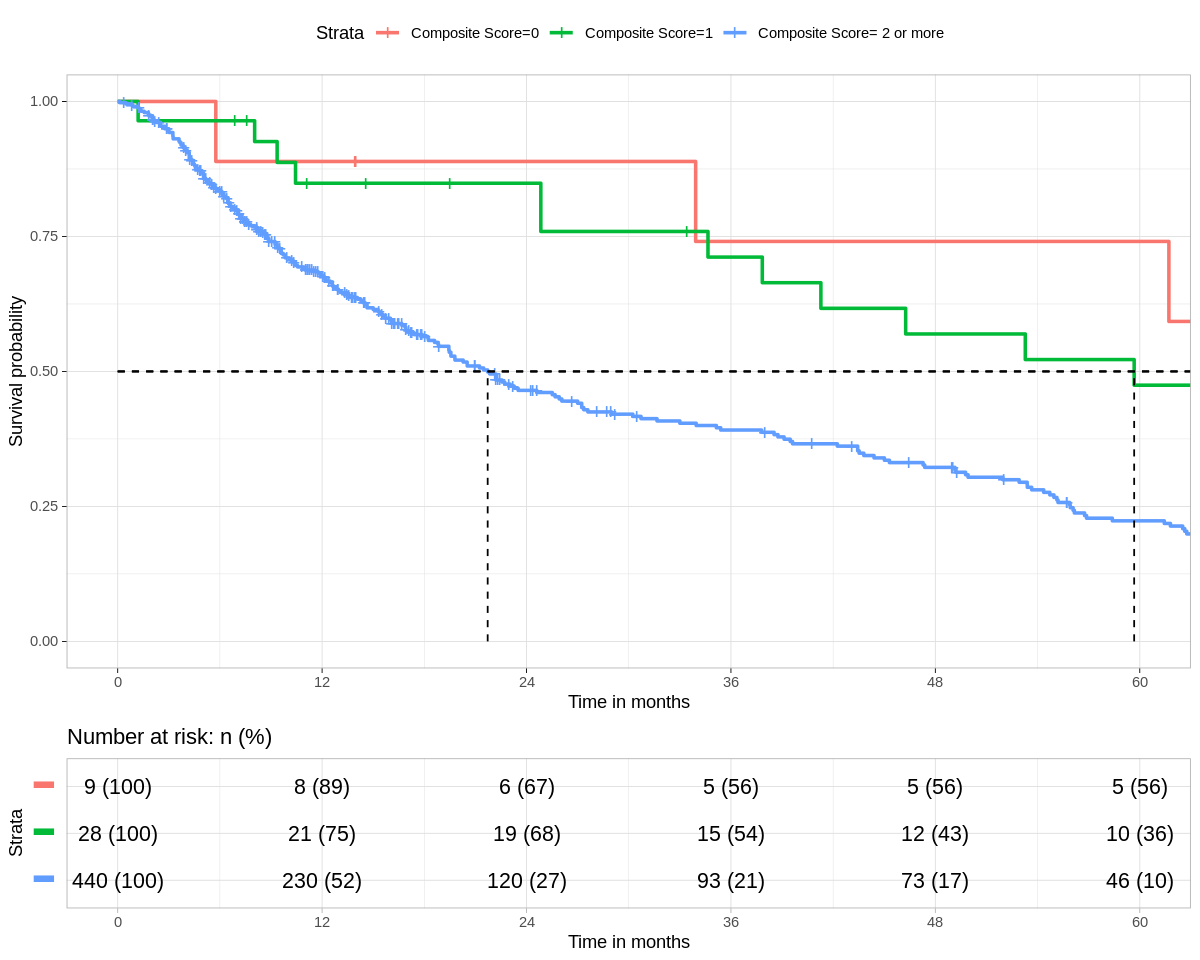


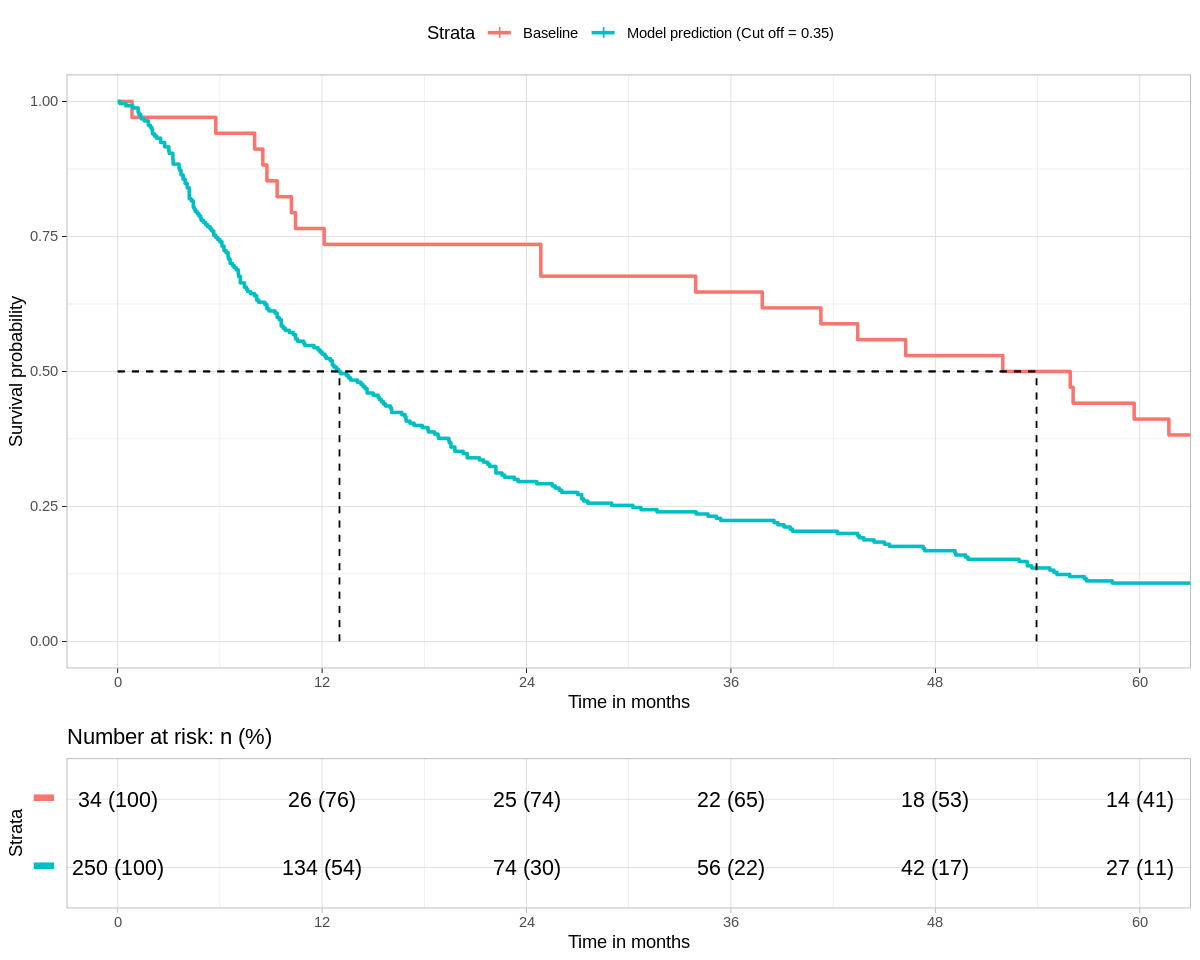

Supplement: Supplementary file 1 [file DataSheet_1.docx]
